# Correlation between the hemoglobin-to-hematocrit ratio and 3-month outcomes in patients with acute ischemic stroke: a secondary analysis based on a prospective cohort study

**DOI:** 10.3389/fneur.2025.1616847

**Published:** 2025-08-13

**Authors:** Dulin Liu, Hongjuan Liu, Yanling Li, Quan Zhou, Zhihua Huang

**Affiliations:** ^1^Medical School, Hunan University of Chinese Medicine, Changsha, China; ^2^Changde Hospital, Xiangya School of Medicine, Central South University, Changde, China

**Keywords:** hemoglobin, hematocrit, stroke, adverse outcomes, cohort study

## Abstract

**Background:**

Our study dedicated to clarify the connection between the hemoglobin (HB) to hematocrit (HCT) (HB/HCT) ratio and adverse outcomes (AOs) in patients with acute ischemic stroke (AIS) within 3 months.

**Methods:**

A total of 1,906 individuals with AIS were encompassed at a hospital in South Korea. A binary logistic regression model (BLRM) was utilized to explore the linear relationship between the HB/HCT ratio and AOs in patients with AIS. Additionally, generalized additive models (GAMs) and smooth curve fitting were leveraged to investigate the nonlinear relationship between the HB/HCT ratio and AOs in patients with AIS. Two steps recursive method was adopted to calculate the inflection point.

**Results:**

There was a nonlinear relationship between the HB/HCT ratio (per 0.01) and AOs in AIS patients according to the GAMS score and smooth curve fitting. The inflection point of the HB/HCT ratio (per 0.01) is 33.191. On the left side of the inflection point, the risk of AOs decreased by 27.80% for every 1 unit increase in the HB/HCT ratio (per 0.01) (odds ratio (OR) = 0.722, 95% confidence interval (CI): 0.577, 0.903, *p* = 0.004). On the right side of the inflection point, the risk of AOs increased by 20.90% for every 1 unit increase in the HB/HCT ratio (per 0.01) (OR = 1.209, 95% CI: 1.014, 1.440, *p* = 0.034).

**Conclusion:**

Our study revealed a U-shaped relationship between the HB/HCT ratio and the prognosis of AIS. Monitoring HB/HCT ratio may be able to identify patients with AOs in advance, ultimately providing more reference of scientific and effective care for AIS patients which may be linked to the improvement of their clinical outcomes.

## Introduction

Stroke, including ischemic stroke and hemorrhagic stroke, influences 13.7 million people worldwide each year (1). It is the second precipitating factor of disability and death globally, especially ischemic stroke (2). Simultaneously, the disease imposes a significant economic strain on patients, particularly in low-and middle-income countries, where the burden is the heaviest (2). It is estimated that one in five people will experience a stroke in their lifetime in some high-income countries, and in low-income countries, the population who experiences stroke is almost one in two (1). These stroke survivors are at high risk, so the improvement of secondary prevention strategies for the population are critical. Biomarkers in blood may provide more information on the identified prognostic factors, thereby effectively improving the prognosis of stroke patients (3, 4). However, the prognosis of AIS patients remains challenging due to the limitations in the prediction of existing models. In previously identified studies, certain clinical biomarkers, such as natriuretic peptide, copeptin, procalcitonin, mannose-binding lectin, adipocyte fatty acid binding protein, cortisol, were associated with the prognosis of AOs in AIS patients (5). However, the above-mentioned indicators are not the ones routinely used in clinical practice, and they are not easy to obtain. So, both our team and other researchers have been actively seeking easily obtainable, inexpensive, and reliable biomarkers to accurately predict AOs in patients with AIS (3, 4, 6–8).

HB is an important indicator of the prognosis of AIS patients (9). Anemia is very common in stroke patients, with a prevalence of 15–29% (10). A previous study reported a U-shaped association between HB levels and both mortality and functional impairment (9).

HCT is a widely available, simple and inexpensive laboratory test (11) used to assess the effects of HCT on blood viscosity, oxygen-carrying capacity, and hemodynamic stability, all of which may affect cerebral perfusion and tissue oxygenation during ischemic stroke. Studies have shown that a high HCT value is significantly and independently correlated with reduced reperfusion and increased infarct size after ischemic stroke; that is, a high HCT is not conducive to the prognosis of AIS patients (12, 13). Other studies have shown that a low HCT is not conducive to the prognosis of AIS patients (14). Therefore, the relationship between HCT and the prognosis of AIS patients is still controversial.

In the literature, HB/HCT has been confirmed as an effective prognostic marker for polycythemia vera (15), pulmonary embolism (16) and femoral Head Osteonecrosis in Children With Sickle Cell Disease (17). Because polycythemia vera and pulmonary embolism are both related to thrombosis (15, 16), the basis of osteonecrosis of the femoral head in sickle cell disease is related to hemorheology and vascular occlusion (17), and thrombosis, hemorheology changes and vascular occlusion are all related to the onset of acute ischemic stroke, therefore, we suspect that HB/HCT may also be a suitable marker for AIS. However, to the best of our knowledge, no previous studies have explore the association between the HB/HCT ratio and prognosis in AIS patients. Therefore, in this study, we focused on elucidating the relationship between the HB/HCT ratio and AOs in AIS patients.

By clarifying the prognostic value of the HB/HCT ratio in AIS, We hope to provide a scientific basis for early risk stratification and more targeted clinical management, which may ultimately be linked to the improvement of patients outcomes.

## Materials and methods

### Study design

This cohort accurately compiled data from the exclusive prospective registration system established in South Korea, with a time ranging from January 2010 to December 2016 (18). The primary independent variable was the HB/HCT ratio (per 0.01), and the dependent variable was the 3-month clinical outcomes of AIS patients, categorized as AOs or good outcomes.

### Data source

The data were compiled from the open-access study titled “Geriatric Nutritional Risk Index Predicts AOs in AIS Patients-Automated Undernutrition Screen Tool” by Kang et al. (18). This article is distributed under a Creative Commons attribution license, which permits unrestricted use, distribution, and reproduction in any medium, provided the original author and source are credited (18).

### Study population

**Figure 1:**
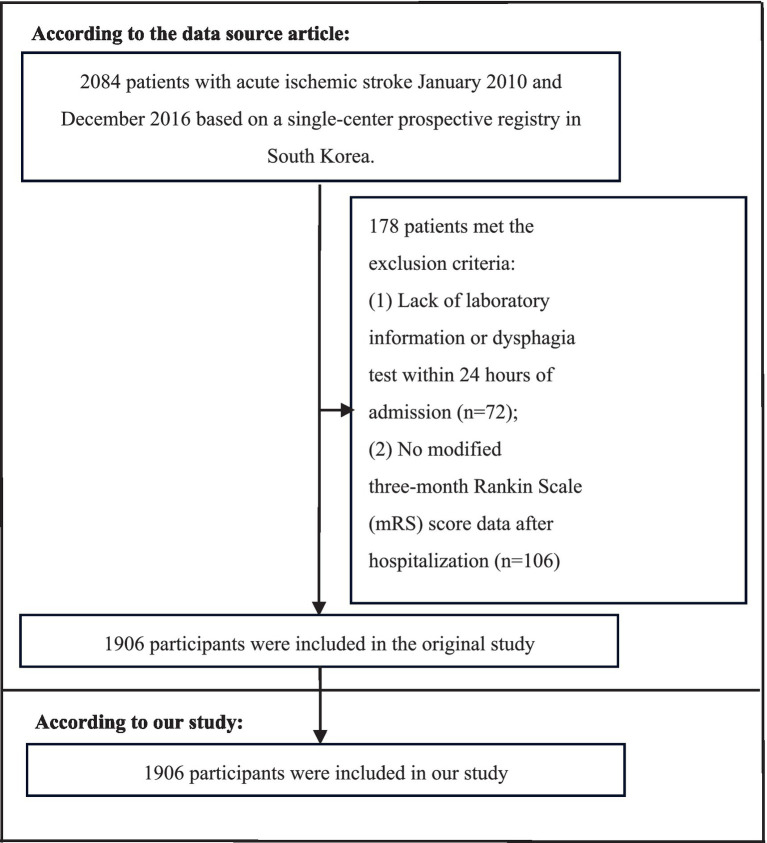
Flowchart of patient selection and exclusion in the original study and our study.

Utilizing data compiled from a Korean single-center prospective registry initiated in October 2002, we included 2,084 patients who were admitted within 7 days of symptom onset and diagnosed with AIS between January 2010 and December 2016. In accordance with hospital regulations, it was assumed that blood samples were collected at the time of the first blood draw upon admission. (1) patients who lacked dysphagia testing or correlated laboratory information within 24 h of admission; (2) patients whose modified Rankin Scale (mRS) score was not recorded 3 months post-stroke. A detailed flowchart outlining the patient selection process is presented in Figure 1. Considering that the original study (18) had already obtained approval from the Institutional Review Board of Seoul National University Hospital (Approval Number: 1009-062-332), no additional ethical approval was required for the subsequent secondary analysis.

### Variable

The HB/HCT ratio (per 0.01) was obtained by dividing the serum HB concentration (g/dL) and the serum HCT (%), making for a continuous variable. The continuous variables were then classified by quartile: Q1: <32.880, Q2: 32.882–33.632, Q3: 33.634–34.353, and Q4: ≥34.354.

### Three-month prognosis in AIS patients

The 3-month prognosis of AIS patients after onset was systematically assessed via the mRS score (19), with data documented through outpatient visits or telephone interviews. It should be emphasized that the structural design of these interviews was standard and reliable (18). Finally, the participants were subdivided into two cohorts in accordance with the outcomes: those with good outcomes, defined as an mRS score of ≤2; and those with AOs, defined as an mRS score of ≥3 (Table 1).

**Table 1:** Incidence rate of AOs at the three-month mark following the occurrence of a stroke.

HB/HCT ratio (per 0.01, %)	Participants (*n*)	Unfavorable outcome events (mRS score ≥ 3)	Incidence of unfavorable outcome (95%CI) (%)
Total	1,906	546	28.65 (26.65–30.71)
Q1	477	170	35.64 (31.43–40.02)
Q2	475	129	27.16 (23.30–31.29)
Q3	477	122	25.58 (21.81–29.64)
Q4	477	125	26.21 (22.41–30.29)

HB, hemoglobin concentration; HCT, hematocrit; mRS, modified Rankin scale; HB/HCT ratio, hemoglobin-to-hematocrit ratio.

### Missing data processing

There were a bunch of different variables on the continuum, Including but not exclusively white blood cell (WBC) count, red blood cell (RBC) count, hemoglobin (HB) level, platelet (PLT) count, hematocrit (HCT), mean red blood cell volume (MCV), mean red blood cell hemoglobin concentration (MCHC), red blood cell distribution (RDW), total cholesterol (TC), serum triglyceride (TG) level, low-density lipoprotein cholesterol (LDL-C) level, high-density lipoprotein cholesterol (HDL-C) level, glutamic-pyruvic aminotransferase (ALT), aspartate aminotransferase (AST), blood urea nitrogen (BUN), serum creatinine (Cr), the glomerular filtration rate (GFR), serum albumin (ALB), total protein (TP), fasting blood glucose (FBG), activated partial thromboplastin time (APTT), fibrinogen (FIB) and body mass index (BMI). In this current study, the occurrences of data missing data for pivotal variables—specifically, HDL-C, FBG, and TC—were observed to be 99 cases (5.19%), 139 cases (7.29%), 1 case (0.00%), respectively. During the data preprocessing stage, we adopted the method of mean imputation to solve the adverse effects of these missing covariates on statistical integrity.

### Statistical analysis

We applied a descriptive method to depict continuous variables, where variables with a Gaussian distribution were articulated in the form of the mean ± standard deviation (SD), and variables with a skewed distribution were articulated in the form of a median (interquartile). We used frequencies and percentages to describe categorical variables. The approach utilized a blend of χ^2^ tests, one-way ANOVA (analysis of variance), and Kruskal–Wallis H tests in order to identify and analyze distinctions among various HB/HCT ratio (per 0.01) groups. We then performed a rigorous collinearity assessment (Supplementary Table 1), Method of selection: We calculated the Variable inflation factor (VIF) for each variable and removed the variable if the highest VIF value was ≥5. At the first collinearity assessment, VIF values were <5 for all variables, and all variables were retained.

In a bid to reveal a significant association between the HB/HCT ratio and AOs in patients with AIS, we conducted univariate analysis and multivariate binary logistic regression analyses. For the multivariate binary logistic regression analyses, we designed three models. The first modeling framework was characterized by an unadjusted model that offered initial insights, subsequently followed by a minimally adjusted model that consisted of selected sociodemographic factors, namely, age and gender. The last model was a fully tuned model. The model completely integrated sociodemographic variables and a wide range of clinical features (including age, sex, RBC, PLT, FBG, BMI, CHD, DM, HT, AF, hyperlipidemia, previous mRS score, previous stroke/TIA, stroke etiology, smoking status and NIHSS score). Using a combination of clinical expertise, previous references and results from univariate analyses, the effect size was accurately described by 95% confidence intervals (CIs).

In order to further verify whether a linear relationship exists, we utilized sensitivity analysis, where we transformed the HB/HCT ratio (per 0.01) into quartiles to thoroughly examine trends via *p*-values. Owing to the remarkable associations between AOs in patients with AIS and DM, abnormal FBG, hyperlipidaemia, elevated BMI, and abnormal Cr (20–22), we excluded those with DM, TC ≥ 200 mg/dL, FBG ≥ 6.1 mmol/L, BMI ≥ 25 kg/m^2^ and Cr ≥ 1.2 mg/dL from the sensitivity analysis.

To disclose the potential nonlinear relationship between the HB/HCT ratio (per 0.01) and AOs, researchers have adopted complicated methods such as generalized additive models (GAMs) and complex smooth curve fitting methods. After the potential nonlinear relationship was found out, we calculated the inflection point by using two steps recursive method. We then set up binary logistic regression models (BLRMs) at both ends of the inflection point. The selection of the most appropriate model depended on serious evaluation through log-likelihood ratio tests, thorough deliberation of categorical influence and diagnostic thresholds.

All results were reported in accordance with the STROBE guidelines (23). Statistical analyses were conducted using R software (http://www.R-project.org, The R Foundation) and EmpowerStats (http://www.empowerstats.com, X&Y Solutions, Inc., Boston, MA). A *p*-value < 0.05 was considered statistically significant.

## Results

### Population characteristics

After conducting a thorough screening process guided by stringent eligibility standards, the study strictly excluded cases where dysphagia testing or necessary laboratory data were insufficient within the critical 24-h window after admission (*n* = 72) and cases where mRS score was not recorded 3 months after stroke (*n* = 106). After excluding 178 cases, the final analysis cohort comprised 1,906 patients. Table 2 portrays the demographic and clinical characteristics of the study cohort. The cohort consisted of 59.59% males (1,168 persons). Participants were subdivided into the following age categories: <60 years (436 people, 22.88%) and ≥60 years (1,470 people, 77.12%). Etiologic classification of stroke: 606 cases (31.79%) of small vessel occlusion (SVO), 365 cases (19.15%) of large artery atherosclerosis (LAA), 493 cases (25.87%) of cardiac embolism (CE), 171 cases (8.97%) of other determined stroke, and 271 cases (14.22%) of undetermined stroke. The median (quartile range) NIHSS score was 3 (0–33). The participants were further subdivided into subgroups on the basis of the HB/HCT ratio (per 0.01) quartiles: Q1: ≤32.878, Q2: 32.880–33.632, Q3: 33.634–34.353, and Q4: ≥34.354. Compared with those of Q1, the HB, TG, HCT and ALT levels of Q4 were increased, while PLT and ESR were decreased. In Q4, the proportion of males (72.537%) was greater. The proportions of patients with NIHSS scores ≥ 14 (8.386%) and patients with AF (16.562%) were lower.

**Table 2:** Participants’ baseline characteristics categorized by the quintiles of the HB/HCT ratio.

Characteristics	HB/HCT-ratio (per 0.01, %)				*p*-value	*p*-value^*^
			
	(25.573–32.880)	(32.882–33.632)	(33.634–34.353)	(34.354–37.453)		
Participants	477	475	477	477		
Demographics						
Age, (years)					<0.001	-
<60	70 (14.675%)	100 (21.053%)	118 (24.738%)	148 (31.027%)		
≥60	407 (85.325%)	375 (78.947%)	359 (75.262%)	329 (68.973%)		
Sex, *n* (%)					<0.001	-
Male	246 (51.572%)	273 (57.474%)	303 (63.522%)	346 (72.537%)		
Female	231 (48.428%)	202 (42.526%)	174 (36.478%)	131 (27.463%)		
Smoking, *n* (%)	157 (32.914%)	165 (34.737%)	202 (42.348%)	226 (47.379%)	<0.001	-
Current smoking, *n* (%)	101 (21.174%)	107 (22.526%)	128 (26.834%)	149 (31.237%)	0.001	-
Medical history						
Hypertension, *n* (%)	320 (67.086%)	301 (63.368%)	290 (60.797%)	300 (62.893%)	0.238	-
DM, *n* (%)	175 (36.688%)	132 (27.789%)	148 (31.027%)	159 (33.333%)	0.027	-
CHD, *n* (%)	66 (13.836%)	65 (13.684%)	42 (8.805%)	47 (9.853%)	0.024	-
AF, *n* (%)	122 (25.577%)	111 (23.368%)	95 (19.916%)	79 (16.562%)	0.004	-
Hyperlipidemia, *n* (%)	166 (34.301%)	180 (37.395%)	182 (38.155%)	171 (35.849%)	0.656	-
Previous stroke/TIA, *n* (%)	128 (26.834%)	81 (17.053%)	101 (21.174%)	92 (19.287%)	0.002	-
Previous mRS, *n* (%)					0.002	-
0 to 2	349 (73.166%)	394 (82.947%)	376 (78.826%)	385 (80.713%)		
≥3	128 (26.834%)	81 (17.053%)	101 (21.174%)	92 (19.287%)		
Clinical features						
BMI (kg/m^2^)	22.955 ± 3.444	23.313 ± 3.135	23.856 ± 3.158	23.868 ± 3.185	<0.001	<0.001
Baseline NIHSS score, *n* (%)					0.012	-
<6	297 (62.264%)	322 (67.789%)	339 (71.069%)	341 (71.488%)		
6 to 13	110 (23.061%)	91 (19.158%)	89 (18.658%)	96 (20.126%)		
≥14	70 (14.675%)	62 (13.053%)	49 (10.273%)	40 (8.386%)		
Laboratory parameters, median (IQR)						
WBC (10^9^/L)	7.600 (2.650–28.110)	7.800 (2.000–21.240)	7.590 (2.430–22.470)	7.740 (1.500–27.000)	0.902	0.754
RBC (10^12^/L)	4.150 (1.580–6.350)	4.350 (2.780–6.540)	4.430 (2.070–6.040)	4.480 (2.250–5.910)	<0.001	<0.001
HB (g/dL)	12.400 (4.800–17.100)	13.600 (8.500–18.400)	14.000 (6.700–18.400)	14.400 (7.300–18.800)	<0.001	<0.001
HCT (%)	38.700 (15.300–53.200)	40.700 (25.500–55.800)	41.300 (19.700–54.400)	41.200 (20.800–54.300)	<0.001	<0.001
MCHC (%)	32.400 (25.600–33.100)	33.300 (32.800–33.700)	34.000 (33.600–34.400)	34.800 (34.300–37.200)	<0.001	<0.001
RDW (%)	13.600 (11.700–28.400)	13.200 (11.200–18.600)	12.900 (11.300–20.200)	12.700 (11.100–20.200)	<0.001	<0.001
MCV (fl)	93.400 (65.900–121.500)	93.400 (78.000–110.500)	92.900 (79.500–110.100)	92.000 (80.900–120.300)	0.004	<0.001
PLT (10^9^/L)	222.000 (20.000–725.000)	218.000 (43.000–649.000)	220.000 (34.000–557.000)	212.000 (23.000–531.000)	0.019	0.216
TC (mg/dL)	169.000 (61.000–448.000)	178.000 (0.000–367.000)	182.000 (67.000–333.000)	181.000 (68.000–330.000)	0.002	<0.001
TG (mg/dL)	94.000 (34.000–355.000)	99.000 (29.000–428.000)	99.000 (29.000–421.000)	105.338 (27.000–450.000)	<0.001	<0.001
HDL-C (mg/dL)	44.165 (12.000–94.000)	45.000 (20.000–87.000)	44.165 (6.000–99.000)	44.000 (11.000–113.000)	0.223	0.060
LDL-C (mg/dL)	97.000 (29.000–355.000)	106.000 (38.000–249.000)	106.000 (18.000–252.000)	105.000 (29.000–241.000)	0.009	0.002
Hyperlipidemia (%)	166 (34.801%)	180 (37.895%)	182 (38.155%)	171 (35.849%)	0.656	-
BUN (mg/dL)	17.000 (4.000–103.000)	16.000 (5.000–93.000)	15.000 (4.000–85.000)	16.000 (4.000–58.000)	<0.001	<0.001
Cr (mg/dL)	0.890 (0.370–11.380)	0.880 (0.360–9.860)	0.880 (0.360–9.860)	0.900 (0.400–13.910)	<0.001	0.451
GFR (%)	74.200 (3.400–237.800)	77.500 (0.000–171.900)	79.400 (0.000–171.200)	80.300 (0.000–170.000)	0.018	0.003
ALT (U/L)	16.000 (2.000–177.000)	18.000 (0.000–167.000)	19.000 (1.000–118.000)	20.000 (3.000–146.000)	<0.001	<0.001
AST (U/L)	22.000 (9.000–218.000)	23.000 (10.000–232.000)	23.000 (9.000–122.000)	23.000 (10.000–208.000)	0.915	0.140
ALB (g/dL)	4.000 (2.100–5.000)	4.100 (2.000–5.000)	4.100 (2.000–5.000)	4.200 (2.100–5.000)	<0.001	<0.001
TP (g/dL)	7.000 (4.500–10.300)	7.000 (5.000–8.600)	7.100 (3.700–8.800)	7.100 (3.700–8.500)	0.036	0.015
FBG (mg/dL)	99.000 (27.000–271.000)	97.000 (34.000–367.000)	99.055 (29.000–414.000)	99.055 (23.000–329.000)	0.156	0.027
FIB (mg/L)	334.000 (125.000–770.000)	320.000 (126.000–697.000)	317.000 (136.000–815.000)	312.000 (128.000–771.000)	<0.001	<0.001
APTT (s)	30.800 (18.000–64.300)	30.400 (20.200–97.100)	30.400 (18.100–61.600)	30.200 (20.200–118.700)	0.574	0.298
Ischemic stroke subtype, *n* (%)					<0.001	-
SVO	127 (26.625%)	149 (31.368%)	166 (34.801%)	164 (34.382%)		
LAA	82 (17.191%)	83 (17.474%)	89 (18.658%)	111 (23.270%)		
CE	151 (31.656%)	133 (28.000%)	112 (23.480%)	97 (20.335%)		
Other determined	59 (12.369%)	36 (7.579%)	40 (8.386%)	36 (7.547%)		
Undetermined	58 (12.159%)	74 (15.579%)	70 (14.675%)	69 (14.465%)		
Unfavorable outcome	170 (35.639%)	129 (27.158%)	122 (25.577%)	125 (26.205%)	0.001	-

The values are as follows: mean ± standard deviation or median (quartile) or number (%); WBC, white blood cell; RBC, red blood cell; HB, hemoglobin concentration; HCT, hematocrit; MCV, corpuscular volume; MCHC, corpuscular hemoglobin concentration; RDW, red blood cell distribution; PLT, platelet; TG, triglyceride; TC, total cholesterol; HDL-C, high-density lipoprotein cholesterol; LDL-C, low-density lipoprotein cholesterol; BUN, blood urea nitrogen; Cr, serum creatinine; GFR, glomerular filtration rate; ALT, alanine aminotransferase; AST, aspartate aminotransferase; ALB, serum albumin; TP, total protein; FBG, fasting blood glucose; FIB, fibrinogen; APTT, activated partial thromboplastin time; BMI, body mass index; DM, diabetes mellitus; CHD, coronary heart disease; AF, atrial fibrillation; TIA, transient ischemia attack; mRS, modified Rankin scale; LAA, large artery atherosclerosis; SVO, small vessel occlusion; CE, cardioembolism; NIHSS, National Institute of Health Stroke Scale; HB/HCT ratio, hemoglobin to hematocrit ratio. p*: nonparametric tests were used when the data did not satisfy normal distribution or homogeneity of variance.

### Three-month incidence of AOs

A total of 546 participants experienced AOs, with an overall incidence of 28.65% (26.65–30.71%). According to the HB/HCT ratio (per 0.01) quartile, the incidence of Q1 was significantly stratified: the incidence of Q1 was 35.64 (31.43–40.02), the incidence of Q2 was 27.16 (23.30–31.29) in the second quartile, and the incidence of Q3 was 25.58 (21.81–29.64). The incidence of Q4 was 26.21 (22.41–30.29). These quartile-specific incidence rates demonstrated a fluctuating risk associated with different HB/HCT ratios, revealing a significant gradient in the likelihood of AOs in the cohort (Table 1).

### Results of the univariate analysis via the BLRM

The univariate analysis clarified that in patients suffering from AIS there was no statistically significant association between AOs and MCV (OR = 0.988, 95% CI: 0.969, 1.007, *p* = 0.219), PLT (OR = 0.999, 95% CI: 0.998, 1.001, *p* = 0.304), CR (OR = 1.017, 95% CI: 0.926, 1.116, *p* = 0.732), GFR (OR = 1.000, 95% CI: 0.996, 1.004, *p* = 0.961), APTT (OR = 0.988, 95% CI: 0.970, 1.007, *p* = 0.205), CHD (OR = 1.025, 95% CI: 0.752, 1.397, *p* = 0.877) or undetermined stroke etiology (OR = 0.875, 95% CI: 0.629, 1.218, *p* = 0.428). In contrast, there were significant differences in age ≥ 60 years (OR = 1.921, 95% CI: 1.478, 2.497, *p* < 0.001), female (OR = 1.660,95% CI: 1.357, 2.030, *p* < 0.001), WBC (OR = 1.079, 95% CI: 1.043, 1.116, *p* < 0.001), RDW (OR = 1.232, 95% CI: 1.154, 1.316, *p* < 0.001), BUN (OR = 1.026, 95% CI: 1.006, 1.027, *p* = 0.002), FBG (OR = 1.008, 95% CI: 1.005, 1.010, *p* < 0.001), FIB (OR = 1.003, 95% CI: 1.002, 1.004, *p* < 0.001), HT (OR = 1.343, 95% CI: 1.088, 1.658, *p* = 0.006), DM (OR = 1.446, 95% CI: 1.174, 1.781, *p* = 0.001), AF (OR = 2.001, 95% CI: 1.590, 2.517, *p* < 0.001), Previous stroke = 3 (OR = 2.110, 95% CI: 1.387, 3.211, *p* = 0.002), NIHSS score at admission 6 to 13 (OR = 6.557, 95% CI: 5.089, 8.450, *p* < 0.001), NIHSS score at admission ≥ 14 (OR = 17.138, 95% CI: 12.223, 24.030, *p* < 0.001), stroke etiology CE (OR = 1.496, 95% CI: 1.156, 1.935, *p* = 0.002) and Other determined stroke etiology (OR = 2.073, 95% CI: 1.458, 2.948, *p* < 0.001) were positively correlated. And smoking (OR = 0.608, 95% CI: 0.493, 0.751, *p* < 0.001), current smoking (OR = 0.779, 95% CI: 0.616, 0.985, *p* = 0.037), BMI (OR = 0.915, 95% CI: 0.886, 0.945, *p* < 0.001), RBC (OR = 0.583, 95% CI: 0.498, 0.682, *p* < 0.001), MCHC (OR = 0.868, 95% CI: 0.797, 0.947, *p* = 0.001), TC (OR = 0.995, 95% CI: 0.993, 0.998, *p* < 0.001), TG (OR = 0.996, 95% CI: 0.994, 0.998, *p* < 0.001), LDL-C (OR = 0.996, 95% CI: 0.993, 0.999, *p* = 0.003), HB (OR = 0.819, 95% CI:0.779, 0.862, *p* < 0.001), HCT (OR = 0.932, 95% CI:0.916, 0.949, *p* < 0.001) and ALB (OR = 0.274, 95% CI: 0.215, 0.350, *p* < 0.001), TP (OR = 0.674, 95% CI: 0.573, 0.793, *p* < 0.001), LAA (OR = 0.622, 95% CI: 0.452, 0.856, *p* = 0.004) and HB/HCT ratio (per 0.01) (OR = 0.870, 95% CI: 0.798, 0.948, *p* = 0.002) showed a negative correlation (Table 3).

**Table 3:** Determinants of AOs in AIS patients assessed through univariate regression analysis.

Variable	Characteristics	OR 95%CI	*p*
WBC (10^9^/L)	8.137 ± 2.887	1.079 (1.043, 1.116)	<0.001
RBC (10^12^/L)	4.323 ± 0.641	0.583 (0.498, 0.682)	<0.001
HB (g/dL)	13.478 ± 2.004	0.819 (0.779, 0.862)	<0.001
HCT (%)	40.072 ± 5.591	0.932 (0.916, 0.949)	<0.001
MCHC (%)	33.598 ± 1.148	0.868 (0.797, 0.947)	0.001
RDW (%)	13.396 ± 1.536	1.232 (1.154, 1.316)	<0.001
MCV (fl)	92.956 ± 5.217	0.988 (0.969, 1.007)	0.219
PLT (10^9^/L)	223.613 ± 71.310	0.999 (0.998, 1.001)	0.304
TC (mg/dL)	179.358 ± 43.759	0.995 (0.993, 0.998)	<0.001
TG (mg/dL)	110.755 ± 54.578	0.996 (0.994, 0.998)	<0.001
HDL-C (mg/dL)	46.459 ± 13.261	0.997 (0.990, 1.005)	0.459
LDL-C (mg/dL)	105.823 ± 38.961	0.996 (0.993, 0.999)	0.003
BUN (mg/dL)	17.599 ± 8.881	1.016 (1.006, 1.027)	0.002
Cr (mg/dL)	1.088 ± 1.037	1.017 (0.926, 1.116)	0.732
GFR (%)	77.874 ± 27.582	1.000 (0.996, 1.004)	0.961
ALT (U/L)	26.107 ± 14.340	1.009 (1.002, 1.016)	0.009
AST (U/L)	22.384 ± 16.098	0.993 (0.986, 1.000)	0.041
ALB (g/dL)	4.019 ± 0.428	0.274 (0.215, 0.350)	<0.001
TP (g/dL)	7.011 ± 0.611	0.674 (0.573, 0.793)	<0.001
FBG (mg/dL)	106.278 ± 36.882	1.008 (1.005, 1.010)	<0.001
APTT (s)	31.121 ± 5.730	0.988 (0.970, 1.007)	0.205
FIB (mg/L)	334.141 ± 84.714	1.003 (1.002, 1.004)	<0.001
BMI (kg/m^2^)	23.498 ± 3.253	0.915 (0.886, 0.945)	<0.001
HB/HCT ratio (per 0.01, %)	33.600 ± 1.153	0.870 (0.798, 0.948)	0.002
SEX, *n* (%)			
Male	1,168 (61.280%)	1.0	
Female	738 (38.720%)	1.660 (1.357, 2.030)	<0.001
Age (years), *n* (%)			
<60	436 (22.875%)	1.0	
≥60	1,470 (77.125%)	1.921 (1.478, 2.497)	<0.001
Hypertension, *n* (%)			
No	695 (36.464%)	1.0	
Yes	1,211 (63.536%)	1.343 (1.088, 1.658)	0.006
DM			
No	1,292 (67.786%)	1.0	
Yes	614 (32.214%)	1.446 (1.174, 1.781)	0.001
Previous stroke/TIA, *n* (%)			
No	1,504 (78.909%)	1.0	
Yes	402 (21.091%)	1.811 (1.437, 2.283)	<0.001
Previous mRS, *n* (%)			
0			
1	176 (9.234%)		
2	114 (5.981%)	0.622 (0.452, 0.856)	0.004
3	99 (5.194%)	1.496 (1.156, 1.935)	0.002
4	73 (3.830%)	2.073 (1.458, 2.948)	<0.001
5	54 (2.833%)	0.875 (0.629, 1.218)	0.428
Hyperlipidemia, *n* (%)			
No	1,207 (63.326%)	1.0	
Yes	699 (36.674%)	0.788 (0.639,0.972)	0.026
NIHSS score, *n* (%)			
<6	1,299 (68.153%)	1.0	
6 to 13	386 (20.252%)	6.557 (5.089, 8.450)	<0.001
≥14	221 (11.595%)	17.138 (12.223, 24.030)	<0.001
AF			
No	1,499 (78.646%)	1.0	
Yes	407 (21.354%)	2.001 (1.590, 2.517)	<0.001
CHD, *n* (%)			
No	1,686 (88.458%)	1.0	
Yes	220 (11.542%)	1.025 (0.752, 1.397)	0.877
Stroke etiology, *n* (%)			
SVO	606 (31.794%)	1.0	
LAA	365 (19.150%)	0.622 (0.452, 0.856)	0.004
CE	493 (25.866%)	1.496 (1.156, 1.935)	0.002
Other determined	171 (8.972%)	2.073 (1.458, 2.948)	<0.001
Undetermined	271 (14.218%)	0.875 (0.629, 1.218)	0.428
Smoking, *n* (%)			
No	1,156 (60.651%)	1.0	
Yes	750 (39.349%)	0.608 (0.493, 0.751)	<0.001
Current Smoking, *n* (%)			
No	1,427 (74.554%)	1.0	
Yes	485 (25.446%)	0.779 (0.616,0.985)	0.037

The values are as follows: mean ± standard deviation or median (quartile) or number (%); WBC, white blood cell; RBC, red blood cell; HB, hemoglobin concentration; HCT, hematocrit; MCV, corpuscular volume; MCHC, corpuscular hemoglobin concentration; RDW, red blood cell distribution; PLT, platelet; TG, triglyceride; TC, total cholesterol; HDL-C, high-density lipoprotein cholesterol; LDL-C, low-density lipoprotein cholesterol; BUN, blood urea nitrogen; Cr, serum creatinine; GFR, glomerular filtration rate; ALT, alanine aminotransferase; AST, aspartate aminotransferase; ALB, serum albumin; TP, total protein; FBG, fasting blood glucose; FIB, fibrinogen; APTT, activated partial thromboplastin time; BMI, body mass index; DM, diabetes mellitus; CHD, coronary heart disease; AF, atrial fibrillation; TIA, transient ischemia attack; mRS, modified Rankin scale; LAA, large artery atherosclerosis; SVO, small vessel occlusion; CE, cardioembolism; NIHSS, National Institute of Health Stroke Scale; HB/HCT ratio, hemoglobin to hematocrit ratio.

### Results of the multivariate logistic regression analysis using the BLRM

By applying the BLRM, three different models were constructed to explore the correlation between the HB/HCT ratio and the likelihood of AOs in AIS patients. The unadjusted model demonstrated that for every 1 unit increase in the HB/HCT ratio (per 0.01), the risk of AOs decreased by 13.00% (OR = 0.870, 95% CI: 0.798, 0.948, *p* = 0.002), which achieved statistical significance. However, in multivariate logistic regression analysis, there were no statistical significance in the moderately adjusted model and fully adjusted model (*p* > 0.05) (Table 4).

**Table 4:** Examination of the relationship between HB/HCT ratio and AOs in AIS patients after 3 months through diverse models.

Variable	Crude model (OR,95%CI)	*p*	Model I (OR,95%CI)	*p*	Model II (OR,95%CI)	*p*
HB/HCT ratio (per 0.01)	0.870 (0.798, 0.948)	0.002	0.924 (0.844, 1.011)	0.086	0.979 (0.878, 1.091)	0.695
HB/HCT ratio (per 0.01, quartile)						
Q1 (25.573–32.880)	1.0		1.0		1.0	
Q2 (32.882–33.632)	0.673 (0.511, 0.887)	0.005	0.700 (0.527, 0.930)	0.014	0.762 (0.539, 1.077)	0.124
Q3 (33.634–34.353)	0.621 (0.470, 0.820)	<0.001	0.708 (0.531, 0.944)	0.019	0.792 (0.559, 1.123)	0.190
Q4 (34.354–37.453)	0.641 (0.486, 0.846)	0.002	0.767 (0.574, 1.025)	0.073	0.890 (0.627, 1.264)	0.516
P for trend		0.001		0.077		0.550

Crude model: we did not adjust for other covariates. Model I: We adjusted for age and sex. Model II: We adjusted for age, sex, RBC, PLT, FBG, BMI, DM, hyperlipidemia, previous stroke or TIA, hypertension, AF, CHD, stroke etiology, smoking, mRS score before the onset of index events and the NIHSS score.

### Sensitivity analysis

We conducted a series of sensitivity analyses. We first transformed the HB/HCT ratio (per 0.01) from a continuous variable to a categorical variable, utilizing quartiles. And reintegrating the categorically converted HB/HCT ratio (per 0.01) into the model. The crude model (Table 4) revealed a negative correlation between HB/HCT ratio and the AOs in patients with AIS. In Model II (Table 4), Q2 (OR = 0.762, 95% CI: 0.539, 1.077, *p* = 0.695), Q3 (OR = 0.792, 95% CI: 0.559, 1.123, *p* = 0.190), and Q4 (OR = 0.890, 95% CI: 0.627, 1.264, *p* = 0.516) were not statistically significant. This observation suggested that there may be a nonlinear relationship between the HB/HCT ratio and AOs in AIS patients.

In additional iterations of the sensitivity analysis, patients with TC ≥ 200 mg/dL, DM, FBG ≥ 6.1 mmol/L, BMI ≥ 25 kg/m^2^ and Cr ≥ 1.2 mg/dL were selectively excluded. The results showed that, after careful adjustment for confounders, the previously observed linear relationship between the HB/HCT ratio (per 0.01) and the risk of AOs remained statistically insignificant (*p* < 0.05) (Table 5). Therefore, the so-called linear association between the HB/HCT ratio (per 0.01) and the risk of AOs did not hold true either in the general population or in specific subgroups.

**Table 5:** Exploration of the correlation between the HB/HCT ratio and AOs across various sensitivity analyses.

HB/HCT ratio (per 0.01)	OR 95%CI	*p*-value
Model I	0.994 (0.866, 1.140)	0.926
Model II	0.997 (0.879, 1.130)	0.959
Model III	0.914 (0.808, 1.035)	0.156
Model IV	1.376 (0.875, 2.163)	0.167
Model V	0.980 (0.865, 1.109)	0.744

Model I was a sensitivity analysis in participants without DM. We adjusted for age, sex, RBC, PLT, FBG, BMI, hyperlipidemia, previous stroke or TIA, hypertension, AF, CHD, stroke etiology, smoking, mRS score before the onset of index events and the NIHSS score. Model II was sensitivity analysis for participants without a BMI ≥ 25 kg/m*^2^*. We adjusted for age, sex, RBC, PLT, FBG, DM, hyperlipidemia, previous stroke or TIA, hypertension, AF, CHD, stroke etiology, smoking, mRS score before the onset of index events and the NIHSS score. Model III was a sensitivity analysis in participants without a total cholesterol (TC) concentration ≥ 200 mg/L. We adjusted for age, sex, RBC, PLT, FBG, BMI, DM, hyperlipidemia, previous stroke or TIA, hypertension, AF, CHD, stroke etiology, smoking, mRS score before the onset of index events and the NIHSS score. Model IV was a sensitivity analysis in participants without FBG ≥ 6.1 mmol/L. We adjusted for age, sex, RBC, PLT, BMI, DM, hyperlipidemia, previous stroke or TIA, hypertension, AF, CHD, stroke etiology, smoking, mRS score before the onset of index events and the NIHSS score. Model V was sensitivity analysis in participants without CR ≥ 1.2 mmol/L. We adjusted for age, sex, RBC, PLT, FBG, BMI, DM, hyperlipidemia, previous stroke or TIA, hypertension, AF, CHD, stroke etiology, smoking, mRS score before the onset of index events and the NIHSS score. OR, odds ratio; CI, confidence interval; Ref, reference; HB/HCT ratio, hemoglobin to hematocrit ratio.

The sensitivity analysis considered all covariates, including age, sex, RBC, PLT, FBG, BMI, CHD, DM, hyperlipidemia, previous mRS score, previous stroke/TIA, hypertension, AF, stroke etiology, smoking and the National Institutes of Health Stroke Scale (NIHSS) score. Notably, Model I for nondiabetic patients did not include diabetes as an adjusted covariate. Model II for patients with BMI < 25 kg/m^2^ did not include BMI as an adjusted covariate. Model IV for patients with FBG < 6.1 mmol/L did not include FBG as an adjusted covariate.

### Addressing nonlinearity via the GAM

**Figure 2:**
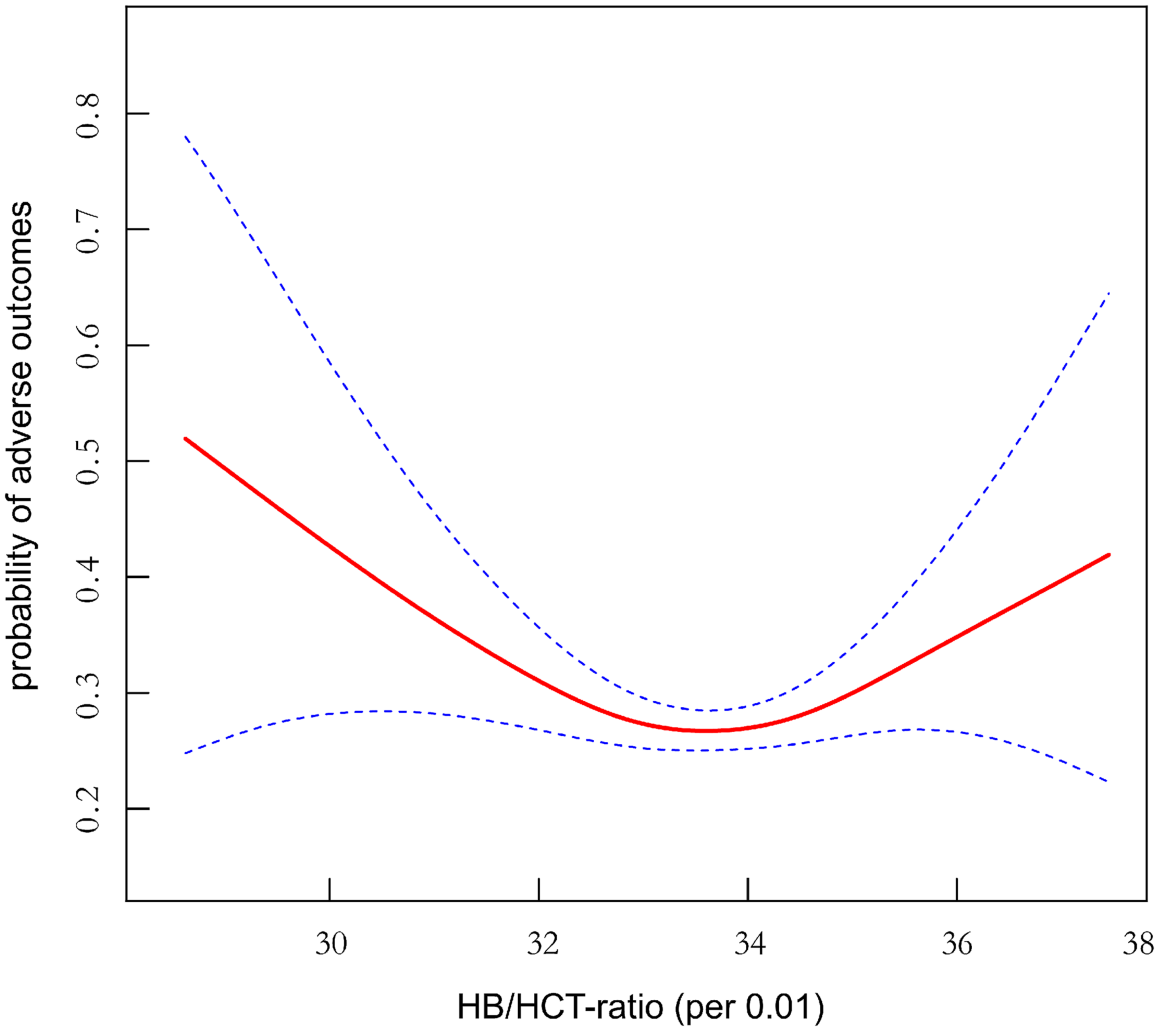
Nonlinear U-shaped association between the HB/HCT (per 0.01) and risk of AOs (mRS ≥ 3) in acute ischemic stroke patients in 3 month.

The results obtained from the multivariable (BLRM) were not statistically significant, indicating the potential influence of intricate nonlinear relationships. What’s more, studies of multivariate adjustment models applying the HB/HCT ratio (per 0.01) quartile as a categorical variable revealed that there was an intricate nonlinear association between the HB/HCT ratio (per 0.01) and AOs in AIS patients. By utilizing sophisticated statistical techniques such as GAM and smooth curve fitting while adjusting a series of synthetic covariates, including age, sex, RBC, PLT, FBG, BMI, CHD, DM, HT, AF, hyperlipidemia, previous mRS score, previous stroke/TIA, stroke etiology, smoking and the NIHSS score, there was a clear U-shaped relationship between the HB/HCT ratio (per 0.01) and AOs in AIS patients (Figure 2). To fully capture the nuances of this relationship, we used a segmented BLRM that accommodates two different slopes, with the model selected on the basis of a log-likelihood ratio test in the sensitivity analysis. Importantly, the *p*-value of the log-likelihood ratio test was <0.05, indicating that the segmented model provided a significantly better fit. With the use of the recursive algorithm, the inflection point was determined to be 33.191. The effect size and CIs on both sides of the inflection point were carefully calculated by applying a two-piecewise BLRM. Especially at the right inflection point, the risk of developing AOs increased by 20.90% for every 1 unit increase in the HB/HCT ratio (per 0.01) (OR = 1.209, 95% CI: 1.014, 1.440, *p* = 0.034). Conversely, on the left side of the inflection point, the effect size (OR) was 0.722 (95% CI: 0.577, 0.903, *p* = 0.004); that is, the risk of developing AOs was reduced by 27.80% for every 1 unit increase in the HB/HCT ratio (per 0.01) (Table 6).

**Table 6:** The outcomes derived from the two-piecewise linear regression model.

Unfavorable outcome	OR (95%CI)	*p*
Fitting model by standard linear regression	0.977 (0.876, 1.090)	0.673
Fitting model by two-piecewise linear regression	33.191	
Inflection point of HB/HCT ratio		
≤33.191	0.722 (0.577, 0.903)	0.004
>33.191	1.209 (1.014, 1.440)	0.034
P for log-likelihood ratio test	0.003	

We adjusted for age, sex, RBC, PLT, FBG, BMI, DM, hyperlipidemia, previous stroke or TIA, hypertension, AF, CHD, stroke etiology, smoking, mRS score before the onset of index events and the NIHSS score.

## Discussion

In this prospective cohort study of 1,906 Korean patients with AIS, the key findings are briefly summarized as follows.

Firstly, the occurrence rate of AOs 3 months after AIS was 28.65%. Secondly, at this three-month mark following AIS, a linear correlation between the HB/HCT ratio and AOs was absent, instead revealing a U-shaped nonlinear relationship. Thirdly, a notable threshold effect was observed, with the HB/HCT ratio demonstrating a turning point at 33.191. At this critical juncture, the incidence of AOs increases linearly when the HB/HCT ratio shifts to extremes (abnormally high or very low). Fourthly, a careful sensitivity analysis was performed to exclude individuals with TC ≥ 200 mg /dL, DM, FBG ≥ 6.1 mmol /L, BMI ≥ 25 kg /m^2^, and Cr ≥ 1.2 mg /dL. Following meticulous adjustment for potential confounding factors, the statistical significance of the linear relationship between the HB/HCT ratio and the risk of AOs remained insignificant, further affirming the persistence of a nonlinear relationship. Our results indicate that a nonlinear U-shaped association between the HB/HCT ratio and AOs at 3 months after AIS, which is an interesting discovery. In this study, an HB/HCT ratio close to 33.191 was associated with a lower incidence of AOs at 3 months after AIS. Therefore, the HB/HCT ratio may be a potential biomarker for predicting stroke prognosis.

Our data provide probably evidence that there remains a U-shaped relationship between the HB/HCT ratio (per 0.01) and AOs in AIS patients. However, its accuracy still requires further verification through future research.

The possible explanations for the relationship between the HB/HCT ratio and the prognosis of AIS patients are as follows: As HB is an important carrier of oxygen, a low HB concentration may lead to a reduction in the tissue oxygen supply. For AIS disease, a reduced oxygen supply is the first crucial step in its occurrence and development. When the infarct area is destroyed, the penumbra area will conduct reperfusion and collateral perfusion spontaneously with the intention of increasing the oxygen supply and further prolonging the resuscitation time. Therefore, low HB may contribute to reducing the oxygen supply in the penumbra area, resulting in necrosis of cells in the penumbra area and expanding the infarct area, which is detrimental to the prognosis of AIS patients (24). Additionally, anemia may also lead to myocardial ischemia and left ventricular hypertrophy, accounting for stroke (25), and expand the infarct size and accelerate its growth (9). Therefore, a low HB concentration maybe closely connected with the prognosis of AIS patients. Moreover, a high HB concentration blood viscosity elevated and cerebral perfusion reduced (25), which may be detrimental to the prognosis of AIS patients.

Low HCT concentrations may result in anemia, reducing the oxygen supply in the penumbra area and promoting the transformation of the penumbra area into the infarction area, which may be detrimental to the prognosis of AIS patients (26). Furthermore, low HCT concentrations may be associated with anemia, malnutrition, renal insufficiency, iron deficiency and inflammation (27), which may also be attributed to AOs in AIS patients. Another point to consider is that a high HCT concentration is closely related to high blood viscosity, which may slow blood flow, reduce cerebral perfusion, and exacerbate penumbral ischemia and hypoxia. In addition to the HCT concentration, red blood cells are essential to the composition of thrombosis (28). A high HCT is associated with the formation of cardiac artery thrombosis (29). High HCT may also indirectly elevate the edge and adhesion of platelets through the activation of adenosine diphosphate (29), increasing the distribution area of platelets on the lower surface of the vascular intima and accelerating the formation of thrombosis (30, 31).

In conclusion, both the HB and HCT levels may result in the AOs of AIS patients. The interaction between HB and HCT is multifaceted. Therefore, utilizing one of these indicators alone to evaluate the prognosis of AIS patients may make the results less accurate and comprehensive.

Additionally, since the HB/HCT ratio we used is a composite indicator, there are different physiological states in patients such as abnormal HB alone, abnormal HCT alone, abnormal both of them and normal both of them. Therefore, utilizing the HB/HCT composite indicator incorporates the simultaneous variations in both HB and HCT, and may be more effective in predicting the prognosis of patients compared to the use of either parameter individually.

Our findings revealed that when the HB/HCT ratio (per 0.01) was near 33.191, the rate of occurrence of AOs in patients with AIS was lower 3 months after AIS. However, what should be noted is that the basic principle of the U-shaped nonlinear relationship between the HB/HCT ratio (per 0.01) and AOs in AIS patients is not clear and needs to be further explored comprehensively.

The limitations of this survey are noteworthy. First of all, our study is based on the secondary analysis of the existing Korean data set. We do not know the data collection process and cannot control the quality of the data, so there may be potential selection bias in the data collection process. Moreover, this data comes from a single center in Korea, which cannot represent the universality. In the future, it is necessary to collect multi-center data for further verification. Secondly, assessing the HB/HCT ratio only at the time of admission, without subsequent assessment during hospitalization, underscores the need to explore potential fluctuations more deeply over time to more fully reveal the dynamics of this biomarker. Thirdly, we did not have any information on the proportion of subjects who received thrombolysis or endovascular therapy, but the small proportion of patients who received these specific treatments had little impact on our results. Fourthly, we did not have any information on the proportion of subjects who regularly received rehabilitation treatment, nor did we assess drug use, which could be an important bias in the study. For example, statin use is strongly associated with stroke outcomes. This is a problem that we should pay attention to in future research. Fifthly, our data are derived from a single-center prospective registration system in South Korea. The blood samples were collected within 0 to 7 days after the onset of stroke symptoms, but the specific collection time is not provided. This wide window period leads to methodological and conceptual flaws, which may affect the accuracy of prognosis assessment.

## Conclusion

In conclusion, this study unveiled a non-linear U-shaped correlation between the HB/HCT ratio and 3-month AOs in AIS patients. When the HB/HCT ratio dipped below 33.191, a remarkable adverse association with AOs was observed. On the contrary, when the HB/HCT ratio exceeded 33.191, a notably positive correlation with AOs emerged. This is helpful to accurately identify patients at higher risk of AOs and provide a reference for clinical decision-making.

## Data Availability

The original contributions presented in the study are included in the article/Supplementary material, further inquiries can be directed to the corresponding authors.
