# Supplementary material for: Correlation between the hemoglobin-to-hematocrit ratio and 3-month outcomes in patients with acute ischemic stroke: a secondary analysis based on a prospective cohort study
Source: Front Neurol. 2025 Aug 13;16:1616847. doi: 10.3389/fneur.2025.1616847 (PMC12380848; doi:10.3389/fneur.2025.1616847)
Supplement: Supplementary file 1 [file Table_1.docx]

***Supplementary*** ***Materials***

1. **Supplementary Figures and Tables**

**1.1 Supplementary Table**

**Supplementary Table 1.** Collinearity screening

|  | Step 1 |
| --- | --- |
| WBC | 1.2 |
| RBC | 2.3 |
| RDW | 1.5 |
| ALB | 2.8 |
| BUN | 2.2 |
| FIB | 1.3 |
| FBG | 1.3 |
| GFR | 2 |
| CR | 2.3 |
| APTT | 1.1 |
| TC | 3.1 |
| TG | 1.2 |
| HDL-c | 1.3 |
| LDL-c | 2.9 |
| MCHC | 1.4 |
| MCV | 1.4 |
| PLT | 1.3 |
| AGE | 1.5 |
| SEX | 1.8 |
| Smoking | 1.9 |
| BMI | 1.3 |
| DM | 1.3 |
| Previous mRS | 1.1 |
| Previous stroke/TIA | 1.1 |
| Hypertension | 1.2 |
| AF | 1.3 |
| CHD | 1.1 |
| NIHSS score | 1.3 |
| Stroke etiology | 1.1 |

Selection method: Calculated VIF for each variable, if highest VIF value >= 5 , remove the variable with highest VIF;

Repeat the above step, until all remaining variables with VIF < 5

NA was the excluded variable

WBC, white blood cell; RBC, red blood cell; RDW, red blood cell distribution; MCV, mean corpuscular volume; PLT, platelet; TG, triglyceride; TC, total cholesterol; HDL-c, high-density lipoprotein cholesterol; LDL-c, low-density lipoproteins cholesterol; BUN, blood urea nitrogen; Cr, serum creatinine; GFR, glomerular filtration rate; APTT, activated partial thromboplastin time; MCHC, corpuscular hemoglobin concentration; ALB, serum albumin; FBG, fasting blood glucose; FIB, fibrinogen; BMI , body mass Index; DM, diabetes mellitus; AF, atrial fibrillation; CHD, coronary Heart Disease; TIA, transient ischemia attack . NIHSS, national Institute of health stroke scale; mRS, modified Rankin scale
